# Dapagliflozin, inflammation and left ventricular remodelling in patients with type 2 diabetes and left ventricular hypertrophy

**DOI:** 10.1186/s12872-024-04022-7

**Published:** 2024-07-12

**Authors:** Adel Dihoum, Alexander JM Brown, Rory J McCrimmon, Chim C Lang, Ify R Mordi

**Affiliations:** 1 Division of Molecular and Clinical Medicine, School of Medicine, University of Dundee, Ninewells Hospital and Medical School, Dundee, Scotland, UK; 2 Division of Systems Medicine, University of Dundee, Dundee, UK

**Keywords:** Sodium-glucose co-transporter 2 inhibitors, Heart failure, Left ventricle hypertrophy, Global longitudinal strain, Inflammation, Cytokines

## Abstract

**Background and Aims:**

Sodium-glucose co-transporter 2 (SGLT2) inhibitors have beneficial effects in heart failure (HF), including reverse remodelling, but the mechanisms by which these benefits are conferred are unclear. Inflammation is implicated in the pathophysiology of heart failure (HF) and there are some pre-clinical data suggesting that SGLT2 inhibitors may reduce inflammation. There is however a lack of clinical data. The aim of our study was to investigate whether improvements in cardiac remodelling caused by dapagliflozin in individuals with type 2 diabetes (T2D) and left ventricular hypertrophy (LVH) were associated with its effects on inflammation.

**Methods:**

We measured C-reactive protein (CRP), tumor necrosis factor alpha (TNF-α), interleukin-1β (IL-1β), interleukin 6 (IL-6), and interleukin 10 (IL-10) and neutrophil-to-lymphocyte ratio (NLR) in plasma samples of 60 patients with T2D and left ventricular hypertrophy (LVH) but without symptomatic HF from the DAPA-LVH trial in which participants were randomised dapagliflozin 10 mg daily or placebo for 12 months and underwent cardiac magnetic resonance imaging (CMR) at baseline and end of treatment. The primary analysis was to investigate the effect of dapagliflozin on inflammation and to assess the relationships between changes in inflammatory markers and LV mass and global longitudinal strain (GLS) and whether the effect of dapagliflozin on LV mass and GLS was modulated by baseline levels of inflammation.

**Results:**

Following 12 months of treatment dapagliflozin significantly reduced CRP compared to placebo (mean difference of -1.96; 95% CI -3.68 to -0.24, *p* = 0.026). There were no significant statistical changes in other inflammatory markers. There were modest correlations between improvements in GLS and reduced inflammation (NLR (*r* = 0.311), IL-1β (*r* = 0.246), TNF-α (*r* = 0.230)) at 12 months.

**Conclusions:**

Dapagliflozin caused a significant reduction in CRP compared to placebo. There were correlations between reductions in inflammatory markers including IL-1β and improvements in global longitudinal strain (but not reduced LV mass). Reductions in systemic inflammation might play a contributory role in the cardiovascular benefits of dapagliflozin.

**Trial registration:**

Clinicaltrials.gov NCT02956811 (06/11/2016).

**Supplementary Information:**

The online version contains supplementary material available at 10.1186/s12872-024-04022-7.

## Introduction

Several large clinical trials involving patients with or without T2D have shown that SGLT2 inhibitors improve cardiovascular outcomes in individuals with heart failure or at risk of heart failure [1, 2]. While the cardiovascular benefits of SGLT2 inhibitors have been consistently demonstrated, the mechanisms by which these benefits are conferred remain unclear. One postulated mechanism is that SGLT2 inhibitors might cause beneficial cardiac remodelling, directly improving cardiovascular structure and function, such as reducing left ventricular hypertrophy (LVH) and improving systolic function [3].

LVH is a marker of cardiovascular risk that is an independent risk factor for future development of heart failure [4]. We and others have previously shown that SGLT2 inhibitors can reduce LV mass compared to placebo, and this might explain some of their cardiovascular benefit in at-risk patients [5]. In the DAPA-LVH trial, we found that 12 months of treatment with dapagliflozin significantly reduced LV mass and improved systolic function measured by GLS compared to placebo in patients with T2D and LVH without symptomatic heart failure. [6, 7]

SGLT2 inhibitors also have cardiometabolic benefits, causing weight loss and improving blood pressure [8]. These favourable changes could lead to a reduction in systemic inflammation that is associated with hypertension and obesity. CRP, a surrogate marker of inflammation, is strongly associated with poor cardiovascular outcomes [9, 10]. Beyond CRP, increased expression of various inflammatory cytokines has been associated with adverse cardiac remodelling including left ventricular hypertrophy, fibrosis, and development of heart failure [11, 12]. Higher levels of systemic inflammation are also associated with adverse outcomes in patients with heart failure. [13, 14]

Several studies have demonstrated that increased levels of inflammation are linked to LVH [15–17]. At a molecular level, elevation in TNF-α triggers the overexpression of pro-inflammatory molecules such as IL-6, a molecular mediator implied in up-regulation of collagen and development of LVH [18–20]. SGLT2 inhibitors have been shown to reduce inflammatory activation. In a preclinical study using a pharmacological model of inflammation in macrophages isolated from patients with diabetes, SGLT2 inhibitors significantly reduced the TNF-α and IL-1β [21].

Given these relationships, it is plausible that at least some of the beneficial cardiac effects of SGLT2 inhibitors might be related to an anti-inflammatory effect, however, this has not been examined in a clinical study in detail. The aim of this study was to evaluate improvements left ventricular remodelling caused by dapagliflozin were associated with its anti-inflammatory effects in patients with T2D and LVH.

## Methods

### Study cohort

The design of DAPA-LVH trial, as well as the study design, protocol and the primary outcomes, have been published previously [22, 23]. In brief, in the trial 66 patients with T2D, LVH, and without uncontrolled hypertension or symptomatic heart failure (i.e. Stage B HF) were randomised to receive either dapagliflozin 10 mg once daily (*n* = 32) or placebo (*n* = 34) for 12 months. DAPA-LVH trial received approval from the East of Scotland Research Ethics Committee (16/ES/0131), and all participants provided written informed consent before enrolment. The trial duration spanned 10–12 months. The primary outcome was a change in absolute left ventricular mass from baseline, assessed by cardiac magnetic resonance imaging. Patients also underwent 2-dimensional transthoracic echocardiography including assessment of GLS.

### Inflammatory marker analysis

Blood samples were obtained at the baseline and 12-month (primary outcome) visits. Samples were centrifuged at 3000 rpm for 10 min then the plasma was carefully decanted into aliquot and subsequently stored in -80⁰C. Plasma levels of CRP were measured using Luminex Human Discovery Assay (1-Plex) LXSAHM-01 and levels of inflammatory cytokines TNF-α, IL-1β, IL-6, and IL-10 were determined using R&D Systems Luminex kits. All experimental measurements were performed according to the manufacturer’s instructions and repeated in duplicate. A full blood count was also obtained, and the eosinophil to lymphocyte ratio (ELR), and neutrophil to lymphocyte ratio (NLR) were measured as an additional markers of systemic inflammation.

### Statistical analyses

Normally distributed continuous variables were reported as mean ± standard deviation while non-normally distributed continuous variables were reported as median with interquartile ranges in parentheses (IQR). Categorical variables were reported as number with percentage of the sample in parentheses. To determine whether differences between the dapagliflozin and placebo groups, a t–test was used for normally distributed continuous variables, a Mann-Whitney *U* test was used for non-normally distributed continuous variables, and a chi-square test was used for categorical variables. An analysis of covariance (ANCOVA) was used to determine whether dapagliflozin caused a significant change in CRP, cytokine levels, ELR, and NLR compared to placebo, adjusting for baseline CRP, cytokine levels, and NLR. We evaluated the correlation between changes in inflammation and changes in LV mass and GLS using the Spearman correlation coefficient. Finally, to determine whether the beneficial effects of dapagliflozin on cardiac remodelling were related to baseline levels of inflammation we divided the cohort by the median level of each inflammatory marker and assessed the effect of dapagliflozin on LVM and GLS, testing for the interaction between groups.

## Results

### Baseline characteristics

60 participants from DAPA-LVH had available plasma samples at baseline and 12 months, matched with echo and CMR studies. The baseline characteristics of the individuals are shown in Table 1. The mean age of the cohort was 65 years and 60% were male. The median CRP was 1.07 mg/L and there were no statistically significant differences in participants randomised to dapagliflozin or placebo. Baseline characteristics stratified by the median CRP at baseline are presented in Supplementary Table 1. Patients with higher levels of CRP were more likely to be female and have a history of stroke, lower LV mass, and have a lower prevalence of hypertension and were less likely to be prescribed angiotensin receptor blockers and statins.

**Table 1:** Baseline data

Variable	Total cohort	Dapagliflozin	Placebo	*p* value
	*n* = 60	*n* = 29	*n* = 31	
**Demographics**				
Age (years)	65.23 ± 6.91	64.14 ± 7.07	66.26 ± 6.72	0.239
Male	36 (60.0%)	18 (62.1%)	18 (58.1%)	0.752
Duration of diabetes (years)	10 (6.0, 15.0)	9 (5.50, 15.00)	10 (5.75, 14.25)	0.582
BMI (kg/m2)	32.58 ± 3.97	32.14 ± 4.34	32.98 ± 3.62	0.418
**Co-morbidities**				
IHD	8 (13.3%)	2 (6.9%)	6 (19.4%)	0.156
Hypertension	47 (78.3%)	24 (82.8%)	23 (74.2%)	0.421
Stroke	7 (11.7%)	1 (3.4%)	6 (19.4%)	0.055
Atrial fibrillation	1 (1.7%)	1 (3.4%)	0 (0.0%)	0.297
Hypercholesterolaemia	38 (63%)	17 (58.6%)	21 (67.7%)	0.464
COPD	2 (3.3%)	1 (3.4%)	1 (3.2%)	0.962
**Medications**				
ACE inhibitor	32 (53.3%)	15 (51.7%)	17 (54.8%)	0.809
Angiotensin receptor blocker	10 (16.7%)	5 (17.2%)	5 (16.1%)	0.908
Calcium channel blocker	21 (35%)	9 (31.0%)	12 (38.7%)	0.533
Beta-blocker	9 (15%)	4 (13.8%)	5 (16.1%)	0.800
Antiplatelet	16 (26.7%)	6 (20.7%)	10 (32.3%)	0.311
Statin	51 (85%)	23 (79.3%)	28 (90.3%)	0.233
Metformin	60 (100%)	29 (100%)	31(100%)	Constant
Insulin	14 (23.3%)	7 (24.1%)	7 (22.6%)	0.887
**Blood pressure**				
24 h SBP	128.46 ± 10.20 (*n* = 59)	129.59 ± 9.25 (*n* = 29)	127.37 ± 11.09 (*n* = 30)	0.408
24 h DBP	73.51 ± 6.75 (*n* = 59)	73.93 ± 8.11 (*n* = 29)	73.10 ± 5.23 (*n* = 30)	0.641
24 h Heart rate	75.76 ± 14.31 (*n* = 59)	75.14 ± 14.21 (*n* = 29)	76.37 ± 14.62 (*n* = 30)	0.745
**Laboratory measurements**				
Haemoglobin (g/L)	138.17 ± 12.91	137.45 ± 13.97	138.84 ± 12.034	0.681
Creatinine (umol/L)	68.63 ± 18.94	65.17 ± 17.01	71.87 ± 20.332	0.173
HbA1c mmol	61.67 ± 10.47	62.55 ± 10.72	60.84 ± 10.341	0.531
CRP (mg/L) ^a^	1.07 (0.47, 3.04)	0.87 (0.36,3.14)	1.19 (0.52,2.88)	0.492
Eosinophils count x10^9^/L	0.19 (0.13, 0.27)	0.21(0.13, 0.28)	0.18 (0.13, 0.25)	0.488
Eosinophils %	3.09% (2.01%, 3.81%)	2.83% (1.91%, 3.96%)	3.15% (2.04%, 3.44%)	0.716
Neutrophils count x10^9^/L	4.00 (3.00, 4.72)	4.1 (3.40, 5.30)	3.70 (2.60, 4.40)	0.212
Neutrophils %	60.92% (55.76%, 63.85%)	61.22% (56.57%, 64.15%)	59.74% (55.26%, 62.50%)	0.350
Lymphocyte count x10^9^/L	2.00 (1.60, 2.40)	2.00 (1.80, 2.80)	1.90 (1.60, 2.30)	0.218
Lymphocyte %	29.52% (26.86%, 35.99%)	29.54% (26.86%, 35.82%)	29.50% (26.86%, 36.50%)	0.965
ELR	0.09 (0.06, 0.13)	0.10 (0.06, 0.13)	0.09 (0.06, 0.13)	0.945
NLR^a^	2.06 (1.56, 2.38) (*n* = 53)	2.07 (1.66, 2.42) (*n* = 27)	2.02 (1.52, 2.36) (*n* = 26)	0.810
IL-1β (pg/ml) ^a^	0.49 (0.45, 0.52)	0.48 (0.45, 0.52)	0.49 (0.45, 0.52)	0.911
IL-6 (pg/ml ^a^	1.27 (1.13, 1.43)	1.24 (1.14, 1.37)	1.31 (1.11, 1.46)	0.378
IL-10 (pg/ml) ^a^	0.61 (0.54, 0.66)	0.61 (0.54, 0.64)	0.61 (0.56, 0.68)	0.382
TNF-α (pg/ml) ^a^	3.7 (2.05, 5.60,56)	2.92 (1.66, 5.03)	3.82 (2.47, 5.54)	0.176
NT pro BNP (pg/ml) ^a^	247.40 (99.98, 560.56)	189.21 (80.50, 570.90)	291.23 (141.22, 521.70)	0.371
**Cardiac MRI**				
Absolute LV mass (g)	125.03 ± 21.68	124.65 ± 21.52	125.38 ± 22.19	0.897
LVMI Height (g/m)	74.01 ± 10.23	73.91 ± 9.92	74.10 ± 10.67	0.946
EF (%)	71.67 ± 5.91	71.74 ± 5.35	71.61 ± 6.48	0.934
EDV (mLs)	123.45 ± 22.70	124.06 ± 20.18	122.871 ± 25.15	0.840
ESV (mLs)	35.43 ± 10.27	35.60 ± 9.37	35.27 ± 11.21	0.903
Global longitudinal strain (%)	-17.80 ± 2.08 (*n* = 45)	-17.75 ± 2.14 (*n* = 23)	-18.25 ± 2.04 (*n* = 22)	0.422

Data are mean ± SD, n (%)

IHD, ischaemic heart disease; MRA, Mineralocorticoid receptor antagonists; EDV, end-diastolic volume; EF, ejection fraction; ESV, end-systolic volume; LV, left ventricular; LVM, left ventricular mass; LVMI, left ventricular mass index; MRI, magnetic resonance imaging; DBP, diastolic blood pressure; SBP, systolic blood pressure; GFR, glomerular filtration rate; CRP, C-reactive protein; NLR, neutrophil-lymphocyte ratio; TNF-α, Tumor necrosis factor-α ; IL-1β, Interleukin-1 beta; IL-6, Interleukin 6; IL-10, Interleukin 10; NT-proBNP, N-terminal pro natriuretic peptide

^a^Median (quartile 1, quartile 3)

### Effects of dapagliflozin on inflammatory markers

After 12 months of treatment, dapagliflozin significantly reduced CRP compared to placebo (Table 2). The change in CRP in the dapagliflozin group was 1.07 ± 0.61 mg/L vs. placebo group 3.04 ± 0.59 mg/L; *p* = 0.026), leading to an absolute mean difference of -1.96 (95% confidence interval (CI): -3.68 to -0.24, *p* = 0.026). Dapagliflozin did not cause any significant changes in TNF-α, IL-1β, IL-6, IL-10, ELR, or NLR compared to placebo.

**Table 2:** Change in inflammatory markers after 12 months of dapagliflozin treatment

Variable	Dapagliflozin (*n* = 29)	Placebo (*n* = 31)	Mean Difference (95%CI)	*P*-value
CRP mg/L	1.07 ± 0.61	3.04 ± 0.59	-1.96 (-3.68 to -0.24)	**0.026**
Eosinophils count x10^9^/L	0.023 ± 0.08	0.155 ± 0.08	-0.13 (-0.38 to 0.12 )	0.299
Eosinophils %	0.14% ± 1.00%	1.96% ± 1.00	-1.82% (-4.69–1.04%)	0.208
Neutrophils count x10^9^/L	-0.00 ± 0.22	-0.10 ± 0.22	0.09 (-0.54 to 0.72)	0.769
Neutrophils %	0.33% ± 1.75%	-0.77% ± 1.75%	1.11% (-3.88–6.10%)	0.657
Lymphocyte count x10^9^/L	-0.09 ± 0.06	-0.056 ± 0.06	-0.04 (-0.22 to 0.13)	0.636
Lymphocyte %	-0.65% ± 0.95%	-0.54% ± 0.95%	-0.11% (-2.82–2.58%)	0.932
ELR	-0.00 ± 0.03	0.06 ± 0.03	-0.06 (-0.18 to 0.04)	0.213
NLR*	0.12 ± 0.11	-0.02 ± 0.11	0.14 (-0.18 to 0.47)	0.375
TNFα pg/ml	1.42 ± 0.20	1.75 ± 0.19	-0.33 (-0.90 to 0.24)	0.253
IL-1β pg/mL	-0.47 ± 6.15	8.74 ± 5.94	-9.22 (-26.39 to 7.95)	0.287
IL-6 pg/mL	0.00 ± 0.06	-0.03 ± 0.05	0.04 (-0.13 to 0.21)	0.639
IL-10 pg/ml	0.10 ± 0.01	0.15 ± 0.01	-0.04 (-0.08 to 0.00)	0.068

Data are mean ± St. Error

*P*-values in bold indicates *p* < 0.05; ˄Absolute mean Difference between groups. * 27 individuals had available NLR measurements in the dapagliflozin group and 26 in the placebo group

### Association between changes in inflammation and left ventricular mass and function

Overall, after 12 months of treatment, compared to placebo, dapagliflozin caused a significant reduction in LV mass (change in LVM: dapagliflozin − 4.61 ± 0.89 g vs. placebo − 0.87 ± 0.86 g; mean difference − 3.74 g, 95% CI: -6.24 to -1.24; *p* = 0.004) and improvement in GLS (change in GLS: dapagliflozin − 1.63% ± 0.44% vs. placebo group − 0.31% ± 0.45%; mean difference − 1.32%, 95% CI: -2.59% to -0.04%; *p* = 0.043).

The correlation between change in inflammatory makers at 12 months and change in LV mass and GLS at 12 months are summarised in Table 3. In summary, there were no significant correlations between changes in LV mass at 12 months and changes in CRP (*r* = 0.124) or any other inflammatory markers. However, our study showed a modest relationship between changes in GLS at 12 months and changes in NLR (*r* = 0.311), TNF-α (*r* = 0.230), and IL-1β (*r* = 0.246).

**Table 3:** Relationship between change in inflammatory markers and change in LV mass and change in GLS

Variable	Correlation with Change in LV Mass at 12 Months (*r*)	Correlation with Change in Global Longitudinal Strain at 12 Months (*r*)
CRP mg/L	0.124	0.050
ELR	0.003	-0.070
NLR	-0.129	0.311
IL-1β pg/ml	-0.026	0.246
IL-6 pg/ml	0.035	0.165
IL-10 pg/ml	-0.016	0.223
TNF-α pg/ml	0.086	0.230

### Impact of baseline levels of inflammation on the effect of dapagliflozin on left ventricular mass and systolic function

**Fig. 1:**
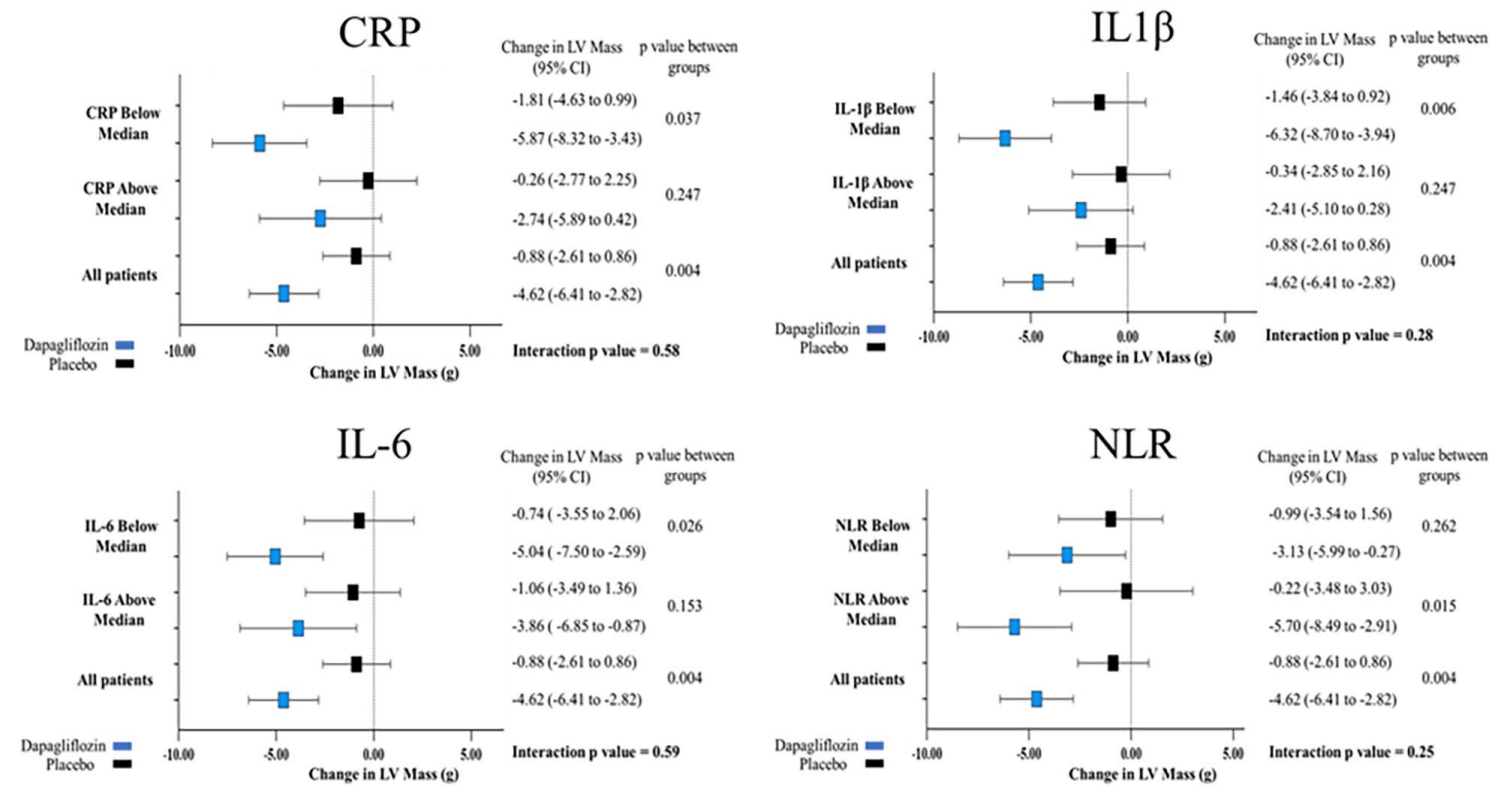
Treatment and 12 months change in left ventricular mass stratified by above and below the median of baseline CRP of 1.07 mg/L, baseline IL-1β of 0.49 pg/ml, baseline IL-6 of 1.26 pg/ml, and baseline NLR of 2.06 in DAPA-LVH trial. Data are presented as mean [95% confidence interval (CI)]. Change in LV mass in grams in the placebo group (black) and the dapagliflozin group (blue)

**Fig. 2:**
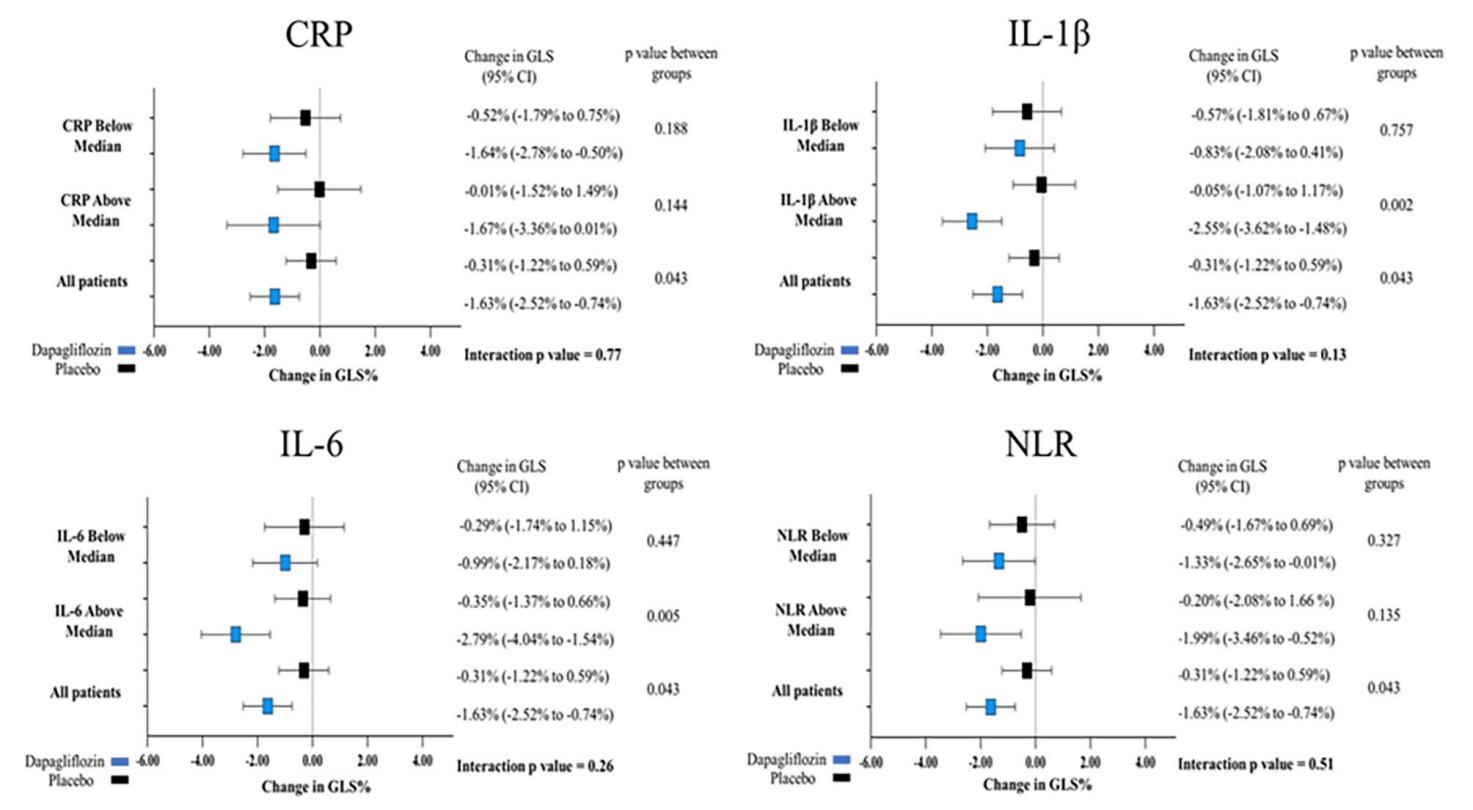
Treatment and 12 months change in global longitudinal strain (GLS) stratified by above and below the median of baseline CRP of 1.07 mg/L, baseline IL-1β of 0.49 pg/ml, baseline IL-6 of 1.26 pg/ml, and baseline NLR of 2.06 in DAPA-LVH trial. Data are presented as mean [95% confidence interval (CI)]. Change in GLS in percentage in the placebo group (black) and the dapagliflozin group (blue)

When the cohort was stratified by the median CRP at baseline there was no significant difference in the effect of 12 months treatment with dapagliflozin on LV mass. In individuals with CRP < 1.07 mg/L, dapagliflozin caused a reduction in LV mass regardless of baseline CRP (mean difference in LV mass with dapagliflozin vs. placebo – CRP < 1.07 mg/L: -5.87 g; 95% CI: 1.19 to -8.32 g, *p* = 0.037; CRP ≥ 1.07 mg/L: -2.47 g; 95% CI: -6.77 to 1.82 g, *p* = 0.247; interaction *p* value = 0.58) (Table 4) (Fig. 1^1^). Results were similar with other inflammatory markers, demonstrating no significant interaction between baseline levels of inflammation and the effect of dapagliflozin on LV mass (Table 4) (Fig. 1). There was also no significant interaction between baseline CRP level and the effect of dapagliflozin on GLS, with dapagliflozin causing similar improvements in GLS regardless of CRP at baseline (mean difference in GLS with dapagliflozin versus placebo – CRP < 1.07 mg/L: -1.12%; 95% CI: -2.83–0.58%, *p* = 0.188; CRP ≥ 1.07 mg/dl: -1.65%; 95% CI: -3.95–0.63%, *p* = 0.144; interaction *p* value = 0.77) (Table 4) (Fig. 2). Furthermore, there was no significant interaction between baseline levels of TNF-α, IL-6, IL-10, ELR, or NLR and the effect of dapagliflozin on GLS (Table 4). Although the interaction *p* value was not statistically significant, there did appear to be a signal that individuals with higher baseline levels of IL-1β might have a greater improvement in GLS with dapagliflozin compared to those with lower levels (mean difference with dapagliflozin versus placebo – IL-1β above median (0.49 pg/ml): -2.61%; 95% CI: -4.17% to -1.04%, *p* = 0.002; IL-1β below median: -0.26%; 95% CI: -2.03–1.50%, *p* = 0.757; interaction *p* value = 0.13) (Fig. 2).

**Table 4:** Effect of Dapagliflozin on LV Mass and GLS stratified by the baseline levels of inflammation

	Dapagliflozin	Placebo	Mean Difference	*P* value	Interaction *P* value
**LV Mass (g)**					
CRP mg/L above median	-2.74 (-5.89 to 0.42)	-0.26 (-2.77 to 2.25)	-2.47 (-6.77 to 1.82)	0.247	
CRP mg/L below median	-5.87 (-8.32 to -3.43)	-1.81 (-4.63 to 1.00)	-5.87 (1.19 to -8.32)	0.037	0.58
ELR above median	-6.01 (-8.61 to -3.41)	-0.64 (-3.55 to 2.25)	-5.36 (-9.27 to -1.45)	0.009	
ELR below median	-2.63 (-5.63 to 0.35)	-0.98 (-3.66 to 1.68)	-1.65 (-5.68 to 2.38)	0.407	0.17
NLR above median	-5.70 (-8.49 to -2.91)	-0.22 (-3.48 to 3.03)	-5.47 (-9.76 to -1.18)	0.015	
NLR below median	-3.13 (-5.99 to -0.27)	-0.99 (-3.54 to 1.56)	-2.13 (-5.98 to 1.70)	0.262	0.25
TNF-α pg/ml above median	-4.67 (-8.12 to -1.23)	0.64 (-2.07 to 3.36)	-5.32 (-9.71 to -0.92)	0.020	
TNF-α pg/ml below median	-4.57 (-6.84 to -2.30)	-2.67 (-5.27 to -0.07)	-1.89 (-5.36 to1.57)	0.272	0.21
IL-1β pg/ml above median	-2.41 (-5.10 to 0.28)	-0.34 (-2.85 to 2.16)	-2.07 (-5.75 to 1.60)	0.257	
IL-1β pg/ml below median	-6.32 (-8.70 to -3.94)	-1.46 (-3.84 to 0.92)	-4.86 (-8.22 to -1.49)	0.006	0.28
IL-6 pg/ml above median	-3.86 (-6.85 to -0.87)	-1.06 (-3.49 to 1.36)	-2.80 (-6.71 to 1.11)	0.153	
IL-6 pg/ml below median	-5.04 (-7.50 to -2.59)	-0.74 ( -3.55 to 2.06)	-4.30 (-8.05 to -0.55)	0.026	0.59
IL-10 pg/ml above median	-3.86 (-7.07 to -0.65)	0.31 (-2.67 to 3.30)	-4.18 (-8.58 to 0.22)	0.062	
IL-10 pg/ml below median	-5.19 (-7.28 to -3.10)	-2.02 (-4.11 to 0.06)	-3.17 (-6.13 to -0.21)	0.037	0.67
**GLS (%)**					
CRP mg/L above median	-1.67 (-3.36 to 0.01)	-0.01 (-1.52 to 1.49)	-1.65 (-3.95 to 0.63)	0.144	
CRP mg/L below median	-1.64 (-2.78 to -0.50)	-0.52 (-1.79 to 0.75)	-1.12 (-2.83 to 0.58)	0.188	0.77
ELR above median	-1.54 (-3.12 to 0.02)	0.30 (-2.22 to 2.84)	-1.85 (-4.84 to 1.13)	0.205	
ELR below median	-2.12 (-3.35 to -0.89)	-0.58 (-1.55 to 0.38)	-1.53 (-3.10 to 0.02)	0.054	0.829
NLR above median	-1.99 (-3.46 to -0.52)	-0.20 (-2.08 to 1.66)	-1.78 (-4.18 to 0.60)	0.135	
NLR below median	-1.33 (-2.65 to -0.01)	-0.49 (-1.67 to 0.69)	-0.84 (-2.61 to 0.93)	0.327	0.514
TNF-α pg/ml above median	-1.68 (-3.33 to -0.04)	-1.02 (-2.77 to 0.72)	-0.66 (-3.10 to1.77)	0.568	
TNF-α pg/ml below median	-1.30 (-2.46 to -0.14)	-0.17 (-1.38 to 1.03)	-1.13 (-2.81 to 0.54)	0.174	0.760
IL-1β pg/ml above median	-2.55 (-3.62 to -1.48)	-0.05 (-1.07 to 1.17)	-2.61 (-4.17 to -1.04)	0.002	
IL-1β pg/ml below median	-0.83 (-2.08 to 0.41)	-0.57 (-1.81 to 0 0.67)	-0.26 (-2.03 to 1.50)	0.757	0.130
IL-6 pg/ml above median	-2.79 (-4.04 to -1.54)	-0.35 (-1.37 to 0.66)	-2.44 (-4.06 to -0.82)	0.005	
IL-6 pg/ml below median	-0.99 (-2.17 to 0.18)	-0.29 (-1.74 to 1.15)	-0.70 (-2.57 to 1.17)	0.447	0.263
IL-10 pg/ml above median	-2.40 (-3.46 to -1.33)	-1.35 (-2.52 to -0.18)	-1.04 (-2.63 to 0.53)	0.183	
IL-10 pg/ml below median	-0.80 (-2.09 to 0.48)	0.56 (-0.67 to 1.79)	-1.36 (-3.15 to 0.43)	0.129	0.625

Data are presented as mean [95% confidence interval (CI)].

## Discussion

In this study, we made several findings. We found that, in patients with T2D and stage B HF, dapagliflozin did significantly reduce CRP compared to placebo, however, it did not lead to significant changes in other markers of inflammation. We also found that the reduction in LV mass caused by dapagliflozin was similar irrespective of baseline levels of inflammation and that changes in LV mass after 12 months of treatment were not correlated with changes in inflammation. In contrast, we did find modest correlations between reductions in NLR, TNF-α, and IL-1β, and improvement in GLS. These results suggest that while any anti-inflammatory effects of dapagliflozin are perhaps not central to its mechanism of benefit, it is possible that reductions in levels of inflammation may contribute to preventing LV systolic impairment, particularly in patients with higher levels of systemic inflammation.

We found a consistent benefit of dapagliflozin on LV mass reverse remodelling regardless of baseline levels of inflammation. This has been reported in other studies, although we have now extended this finding to include other inflammatory markers and CRP. In post hoc analysis of the EMPA-HEART CardioLink-6 trial that included a total of 97 patients with T2D and coronary artery disease (CAD), the effect of empagliflozin on LV mass was stratified by NLR of > 2.1 to evaluate the correlation between baseline NLR and cardiac remodelling. The authors found that the reduction in LV mass caused by empagliflozin was independent of baseline NLR [24], which is consistent with our finding showed that the reduction in LV mass caused by dapagliflozin was not significantly different in patients with T2D and stage B HF, regardless of baseline level of CRP or other inflammatory cytokines. Our study included individuals without CAD and investigated more inflammatory markers CRP, and cytokines, in addition to NLR.

Bozkurt et al. [25] showed an association between elevation of circulating inflammatory makers and deterioration in left ventricular systolic function, finding that there was a stronger correlation between severity of HF and TNF-α or IL-6 levels than with CRP, which was similar to our findings. CRP is a marker of inflammation that is downstream of these cytokines, and it is possible that more upstream markers of inflammation may have more impact on HF outcomes. This hypothesis is also supported by our finding that changes in NLR, a marker of systemic inflammation (as opposed to just one specific pathway) were also more strongly associated with GLS than CRP.

Increased levels of inflammatory cytokines are linked to a reduction in systolic function [26]. Inhibition of IL-1β has been demonstrated to be associated with improvement in LV strain [27] and reduction in heart failure hospitalisation [28]. In our study, we found that reduced IL-1β was correlated with improved subclinical LV systolic function (GLS). Evidence from other studies suggests that this cytokine in particular may be targets for treatment in HF patients, with a post-hoc analysis of CANTOS demonstrating that the IL-1β inhibitor canakinumab caused a dose-dependent reduction in subsequent HF hospitalisation in individuals with high levels of inflammation at randomisation [28]. Although we did not demonstrate a statistically significant reduction in IL-1β with dapagliflozin in our study, we may have been underpowered to do so. Our results are in line with other studies suggesting that IL-1β might be a treatment target for prevention of HF.

### Limitations

Our study is limited by a relatively small sample size and therefore may be underpowered. The study also did not include all inflammatory markers related to cardiovascular disease though we investigated the most common cytokines and markers of inflammation implicated in heart failure.

## Conclusions

In our analysis of the DAPA-LVH trial, we found that in patients with T2D and LVH dapagliflozin significantly reduced CRP but did not cause statistically significant changes in inflammatory cytokines, eosinophils, neutrophils, ELR, and NLR. Although the reduction in LV mass induced by dapagliflozin remained consistent irrespective of the baseline levels of inflammation, there was a modest relationship between higher levels of inflammation at 12 months and worse systolic function. These findings, suggest that while the potential anti-inflammatory effects of dapagliflozin are perhaps not the primary driver of its beneficial mechanism, there is a possibility that these anti-inflammatory effects may contribute to some of the cardiovascular benefits of dapagliflozin, playing a role in prevention of left ventricular systolic impairment, especially in patients with elevated levels of systemic inflammation.

### Electronic supplementary material

Below is the link to the electronic supplementary material.

Supplementary Material 1: Supplementary Table 1-: Baseline characteristics stratified by median CRP (1.07 mg/L) in the DAPA-LVH trial

## Data Availability

The data used and/or analysed during the current study are available from the corresponding author on request.

## Abbreviations

ANCOVA: An analysis of covariance
CAD: Coronary artery disease
CI: Confidence interval
CMR: cardiac magnetic resonance imaging
CRP: C-reactive protein
GLS: Global longitudinal strain
HF: Heart failure
IL-1β: Interleukin-1β
IL-6: Interleukin 6
IL-10: Interleukin 10
LV: Left ventricle
LVH: Left ventricular hypertrophy
TNF-α: Tumor necrosis factor alpha
NLR: Neutrophil-to-lymphocyte ratio
SGLT2: Sodium-glucose co-transporter 2
T2D: type 2 diabetes
