# Supplementary material for: Dapagliflozin, inflammation and left ventricular remodelling in patients with type 2 diabetes and left ventricular hypertrophy
Source: BMC Cardiovasc Disord. 2024 Jul 12;24:356. doi: 10.1186/s12872-024-04022-7 (PMC11241903; doi:10.1186/s12872-024-04022-7)
Supplement: Supplementary file 1 — Supplementary Material 1: Supplementary Table 1-: Baseline characteristics stratified by median CRP (1.07 mg/L) in the DAPA-LVH trial [file 12872_2024_4022_MOESM1_ESM.docx]

Supplementary Table 1. Baseline characteristics stratified by median CRP (1.07mg/L) in the DAPA-LVH trial.

| **Variable** | **CRP < 1.07 mg/L**  **n=30** | **CRP ≥ 1.07 mg/L**  **n= 30** | **P value** |
| --- | --- | --- | --- |
| Baseline CRP (mg/L) | 0.47 (0.27, 0.67) | 2.99 (1.76, 4.42) | **<0.001** |
| **Demographics** |  |  |  |
| Age (years) | 64.73 ± 5.971 | 65.73 ± 7.821 | 0.580 |
| Male | 22 (73.3%) | 14 (46.7%) | **0.035** |
| Duration of diabetes (years) | 10 (8.00, 16.00) | 9 (5.00, 13.25) | 0.124 |
| BMI (kg/m2) | 31.63 ± 2.90 | 33.53 ± 4.67 | 0.064 |
| **Co-morbidities** |  |  |  |
| IHD | 4 (13.3%) | 4 (13.3%) | 1.000 |
| Hypertension | 27 (90.0%) | 20 (66.7%) | **0.028** |
| Stroke | 1 (3.3%) | 6 (20.0%) | **0.044** |
| Atrial fibrillation | 1 (3.3%) | 0 (0.0%) | 0.313 |
| Hypercholesterolaemia | 21 (70.0%) | 17 (56.7%) | 0.284 |
| **Medications** |  |  |  |
| Ace inhibitor | 17 (56.7%) | 15 (50.0%) | 0.605 |
| Angiotensin receptor blocker | 8 (26.7%) | 2 (6.7%) | **0.038** |
| Calcium channel blocker | 13 (43.3%) | 8 (26.7%) | 0.176 |
| Beta-blocker | 5 (16.7%) | 4 (13.3%) | 0.718 |
| Antiplatelet | 7 (23.3) | 9 (30.0%) | 0.559 |
| Statin | 29 (96.7%) | 22 (73.3%) | **0.011** |
| Metformin | 30 (100 %) | 30 (100%) | Constant |
|  |  |  |  |
| Insulin | 5 (16.7%) | 9 (30.0%) | 0.222 |
| **Blood pressure** |  |  |  |
| 24 h SBP | 128.23 ± 9.05  (n=30) | 128.69 ± 11.42  (n=29) | 0.865 |
| 24 h DBP | 74.40 ± 7.20  (n=30) | 72.59 ± 6.25  (n=29) | 0.307 |
| 24 h Heart rate | 70.40 ± 13.32  (n=30) | 81.31 ± 13.33  (n=29) | **0.003** |
| **Laboratory measurements** |  |  |  |
| Haemoglobin (g/L) | 140.43 ± 12.48 | 135.90 ± 13.14 | 0.176 |
| Creatinine (umol/L) | 72.77 ± 17.12 | 64.50 ± 20.04 | 0.091 |
| HbA1c mmol | 59.73 ± 10.48 | 63.60 ± 10.27 | 0.155 |
| Eosinophils count x10^9^/L | 0.19 (0.11, 0.25) | 0.19 (0.14, 0.28) | 0.372 |
| Eosinophils % | 2.93% (1.86%, 3.94%) | 3.22% (2.04%, 3.73%) | 0.603 |
| Neutrophils count x10^9^/L | 3.90 (3.00, 4.40) | 4.00 (2.77, 5.52) | 0.472 |
| Neutrophils % | 59.41% (52.77%, 64.41%) | 61.20% (56.53%, 63.73%) | 0.545 |
| lymphocyte count x10^9^/L | 1.90 (1.75, 2.40) | 2.00 (1.60, 2.50) | 0.821 |
| lymphocyte % | 28.96% (26.75%, 37.04%) | 29.99% (27.03%, 35.44%) | 0.849 |
| ELR | 0.09 (0.04, 0.15) | 0.09 (0.06, 0.13) | 0.533 |
| NLR | 2.03 (1.49, 2.44)  (26) | 2.06 (1.57, 2.32)  (27) | 0.622 |
| IL-1β (pg/ml) | 0.48 (0.46, 0.51) | 0.50 (0.44, 0.55) | 0.807 |
| IL-6 (pg/ml) | 1.17 (1.10, 1.33) | 1.33 (1.24, 1.56) | **0.004** |
| IL-10 (pg/ml) | 0.61 (0.55, 0.65) | 0.60 (0.53, 0.67) | 0.894 |
| TNF-α (pg/ml) | 2.88 (1.53, 5.70) | 3.72 (2.74, 5.07) | 0.225 |
| NT pro BNP (pg/ml) | 221.70 (105.36, 526.53) | 274.42 (85.80, 587.27) | 0.790 |
| **Cardiac MRI** |  |  |  |
| Absolute LV mass (g) | 132.95 ± 21.50 | 117.11 ± 19.09 | **0.004** |
| LVMI Height (g/m) | 77.26 ± 10.13 | 70.76 ± 9.40 | **0.006** |
| EF (%) | 72.48 ± 5.85 | 70.86 ± 5.96 | 0.294 |
| EDV (mLs) | 127.06 ± 23.50 | 119.83 ± 21.66 | 0.220 |
| EDV (mLs) | 127.06 ± 23.50 | 119.83 ± 21.66 | 0.220 |
| ESV (mLs) | 35.33 ± 10.28 | 35.53 ± 10.45 | 0.941 |
| **Echo** |  |  |  |
| Global longitudinal strain (%) (n=45) | -17.97 ± 2.20  (n=27) | -18.03 ± 1.95  (n=18) | 0.931 |

Data are mean ± SD, n (%).

IHD, ischaemic heart disease; MRA, Mineralocorticoid receptor antagonists; EDV, end-diastolic volume; EF, ejection fraction; ESV, end-systolic volume; LV, left ventricular; LVM, left ventricular mass; LVMI, left ventricular mass index; MRI, magnetic resonance imaging; DBP, diastolic blood pressure; SBP, systolic blood pressure; GFR, glomerular filtration rate; CRP, C-reactive protein; NLR, neutrophil-lymphocyte ratio; TNF-α, Tumor necrosis factor-α; IL-1β, Interleukin-1 beta; IL-6, Interleukin 6; IL-10, Interleukin 10; NT-proBNP, N-terminal pro natriuretic peptide.

Median (quartile 1, quartile 3).

Bold indicates p<0.05.
